# Influence of Heat Treatment Parameters on Microhardness of Aluminium Alloy EN AW 7075 Foams and Bulk Material

**DOI:** 10.3390/ma18153562

**Published:** 2025-07-29

**Authors:** Karla Kunac, Nikša Čatipović, Karla Antunović, Damir Jurić

**Affiliations:** Faculty of Electrical Engineering, Mechanical Engineering and Naval Architecture, University of Split, Ruđera Boškovića 32, 21000 Split, Croatia; ncatipov@fesb.hr (N.Č.); karla.perisa.00@fesb.hr (K.A.); damir.juric.03@fesb.hr (D.J.)

**Keywords:** metal foam, heat treatment, microhardness, aluminium alloy EN AW 7075

## Abstract

Aluminium alloy foams have been widely used due to their excellent strength-to-weight ratio, low density, and outstanding properties such as high energy absorption and effective noise and heat insulation. In this study, aluminium machining chips have been used for foam production as a potential recycling method. The process has involved solution heat treatment followed by artificial ageing. Researchers have been analysing the microhardness of both the foam and the bulk material, as well as examining their microstructures. The maximum microhardness value of the bulk material has been found to be 158 ± 2 HV1 at an ageing temperature of 175 ± 1 °C for 2 ± 0.02 h. For the foams, the highest microhardness of 150 ± 2 HV1 has been achieved after ageing at 150 ± 1 °C for 9 ± 0.02 h. Experimental planning has been carried out using Design Expert software. The optimisation process has identified 150 ± 1 °C for 2 ± 0.02 h as the optimum condition for artificial ageing.

## 1. Introduction

Foams have a unique combination of properties [1]. They can be made from a variety of materials, such as metals, composites, ceramics, and polymers. The properties of these types of materials depend on density, porosity, cell size and shape, and homogeneity [2,3]. There are many methods for producing foams. They differ in the way gas is injected or released into the base material [4]. Foams are divided into two main groups. One consists of closed cells, and the other of an open cell structure [5]. The advantage of closed-cell foams is their higher energy absorption [6]. The applications of foams are diverse. They are used as energy absorbers, sound absorbers, filters, and heat exchangers. Foams are also used in many industries such as ship building, construction, automotive, biomedical, and aerospace industries [7]. Aluminium is widely used as a material for foam production. Aluminium foams combine properties such as a lightweight structure, non-toxicity, and high energy absorption [8,9]. They can also be recycled into their base alloy [10]. The 7xxx alloy series consists of the main elements Mg, Zn, and Cu. They combine ductility and strength. This alloy can be subjected to heat treatment, which can improve its properties [11]. It is characterised by low density, high ductility, as well as high strength, toughness and fatigue resistance [12]. The most commonly used foaming agents are calcium carbonate and titanium hydride [13]. Other agents mentioned in the literature are ZrH_2_, dolomite, and magnesium carbonate [14,15].

A major problem in the production of foam is its high cost. One of the reasons for this is the use of expensive metal powder, and another is the use of costly foaming agents [16,17]. Machining chips can be used as a substitute for expensive powders [18]. This method reduces the waste of aluminium alloys [19]. The chips have oxides on their surface [19]. They are also used as stabilisers to increase viscosity. If the viscosity is higher, the melt flows less during foaming. This leads to a higher homogeneity of the final product [20]. If the oxide layer breaks during compaction or melting during recycling, the tensile strength and microhardness of the material increase. The oxide layer has the effect of reducing the deformation properties [19]. The difficulty in using chip waste is the mixing of the foaming agent, which is usually a powder, with larger particles of the base alloy [18].

There is not much research that deals with the production of metal foams from chips [21]. Open-cell structure foams can be made of machining chips by the sintering and dissolution process [22]. The authors compared aluminium foams made from a powder alloy and chips. They concluded that foams with powder addition are more homogeneous and have a higher porosity than the others [18]. Kanetake et al. compared the expansion rate and the pores of foams made from different alloys, EN AW 6063 and EN AW 4032. Alloy EN AW 4032 has a higher expansion rate because it has a lower melting point [23].

The authors produced foams from cans made of the aluminium alloy EN AW 7075 [24]. Another study was conducted with aluminium cans. The main objective was to determine the optimum foaming conditions and the percentage of calcium carbonate. It was concluded that 5 wt.% of calcium carbonate, as well as 800 °C heating temperature and 3 wt.% aluminium oxide, are optimal foaming conditions [25]. It was also investigated how the mechanical properties change with the different particle sizes of the dolomite. It was found that the energy absorption, density, and number of pores decrease with increasing particle size [26].

Another study was concerned with finding the optimum percentage of magnesium addition. The foam was produced with aluminium chips and titanium hydride. Homogeneous pores were formed with the addition of 1 wt.% Mg [17,27].

The authors produced aluminium foams from AlSi_9_Cu_3_ and AlMg_4.5_Mn chips. Foaming agents were calcium carbonate and dolomite at 3 or 5 wt.%. It was found that foams made from dolomite powder exhibit greater expansion. Pores are smaller due to the addition of dolomite [28,29].

Some of the aluminium alloys can be heat-treated. Higher hardness and compressive strength of material have been achieved with the excretion of precipitates, in contrast to steels, in which new phases are formed. Precipitation hardening occurs as a product of heat treatment with three main stages: solution heat treatment, rapid cooling or quenching, and ageing. The first stage is the heating of the material to achieve supersaturation of aluminium with alloying elements, which is accompanied by rapid cooling. The next step is ageing, which can be natural or artificial. The GP zones (Guinier-Preston) are formed [30]. The authors found that heat treatment leads to an improvement in mechanical properties [31,32]. Zuo et al. confirmed this improvement. They also added a nano treatment with TiC particles, which further increased the hardness of the aluminium alloy EN AW 7075 [33]. An increase in mechanical properties can be achieved with cold deformation [34].

Tahmasbi et al. concluded that lower ageing temperatures, such as 120 °C for 24 h, resulted in the highest strength. In contrast, higher temperatures of 180 to 280 °C resulted in a decrease in strength due to grain coarsening [35].

Isadare et al. performed heat treatment of EN AW 7075 alloy in two different ways. The first was annealing at 470 °C, with a holding time of 3 h, and cooling in the furnace. Another method was solution treatment at a temperature of 465 °C for 2 h, quenching, and artificial ageing at 120 °C for 5 h. The authors concluded that the annealed sample had a non-uniform structure and a coarser grain compared to the other. As the sample aged, the yield strength and hardness improved, but the ductility decreased [12].

The aluminium alloy EN AW 7075 was solution treated at 550 °C for two hours, quenched and aged. Artificial ageing was carried out at 190 °C and times of 1 to 20 h. Grain refinement was observed after heat treatment. The microhardness values increased from 76 HV to 160 HV when the time increased from 1 h to 14 h. The authors then concluded that the extended time was too long and that overaging had taken place. In contrast, the yield strength increased from 197.9 MPa at 1 h of ageing to 397.57 MPa at 20 h [11].

Darsono et al. carried out a heat treatment on the aluminium alloy EN AW 7075. The first samples were solution treated at different temperatures, 350, 400, 450, and 500 °C. This process lasted 2 h and continued with a quench of 45 min. Artificial ageing was carried out at 120 °C for 48 h. As the temperature of the solution rose, the microhardness increased from less than 100 HV to 182 HV at 500 °C [36].

There are a few articles about the heat treatment of aluminium foams, and it is a similar process to their base material. After heat treatment, metal foams usually have a higher strength. In some cases, the microhardness can increase by 80%, as in the article [37] for the alloys EN AW 6082 and EN AW 7020. Two samples were quenched in water after foaming, while the others were air-cooled. After water quenching, one of the samples was artificially aged for 12 h at a temperature of 165 °C, while the other sample was not subjected to any heat treatment. The third sample was solution-treated at 530 °C for 100 min, and aged at 165 °C for 12 h, while the fourth sample underwent no heat treatment. It can be concluded that the cooling rate has no significant effect on the hardness properties. The sample with the highest hardness was air-cooled and then heat-treated [30].

Another author proved that the samples had a finer grain after heat treatment, and a significant increase in energy absorption was visible [38]. In another article, it was shown that the energy absorption of the treated sample increased by 40% and the plateau stress doubled during the dynamic compression test [39]. The aluminium alloy EN AW 2014 was foamed and heat-treated. One of the samples was solution treated at 500 °C for 2 h and artificially aged at a temperature of 160 °C for 18 h. Another sample was naturally aged for 5 days after solution treatment. The third sample was not subjected to any heat treatment. It was found that the highest compressive strength is achieved with artificial aging [40].

Jeenager et al. carried out an investigation of the mechanical properties after heat treatment. The samples were foamed at 750 °C. After that, they were aged at temperatures of 100, 150, and 180 °C, and times of 20, 40, 60, and 120 min. The hardness test showed that the maximum microhardness of 122 HV was achieved during the 40-min ageing at 150 °C, compared to the initial sample, where it was 96 HV. Other properties, such as yield stress, plateau stress, and energy absorption, are also improved with increasing temperature and duration of heat treatment [41].

The main aim of this work was to investigate and compare the differences between the microhardness of foams and the base alloy after heat treatment. The heat treatment consists of a solution treatment and artificial ageing.

## 2. Materials and Methods

The aluminium alloy EN AW 7075 was used in this study, Table 1. The supplier for this material was Impol 2000 d.d (Slovenska Bistrica, Slovenia). It was chosen because it has a high potential for use in various industries due to its good mechanical properties. The main advantage of this alloy is its high strength-to-density ratio. For this reason, it is used in the automotive, ship building, and aerospace industries [42]. The main alloying element is Zn. Another advantage of this alloy is its low price compared to other materials with desirable properties, and the possibility of hardening during heat treatment, which further improves the mechanical properties [42]. Chemical composition of the alloy EN AW 7075 is shown in Table 1 [43].

Two different groups of samples were produced. The first group consisted of the solid aluminium alloy EN AW 7075. The number of samples from this group was 10. The other samples were aluminium foams made of the chips of the same alloy, with the same number of samples. A total of 20 samples were prepared.

**Figure 1:**
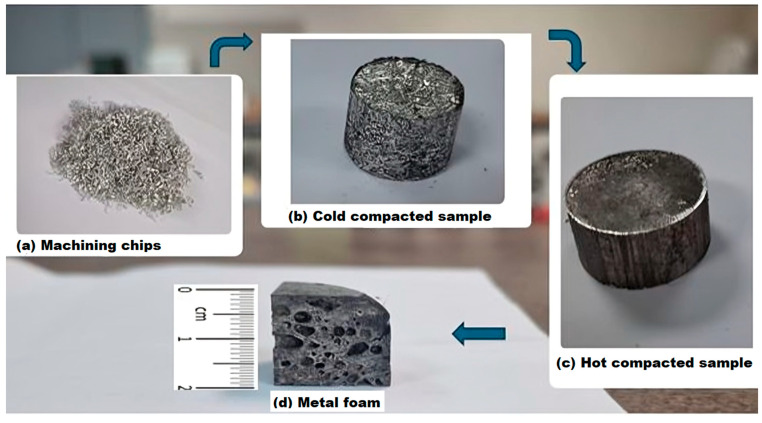
Process of foam production.

The production of metal foams began with the preparation of the machining chips. The Spinner VC 560 vertical machining (SPINNER Werkzeugmaschinen, Sauerlach, Germany) centre was used to produce the chips, Figure 1a. The samples were not lubricated, and dry face milling was used to avoid contamination. Chips were then mixed with the 0.5 ± 0.1 wt.% foaming agent TiH_2_. The next step was cold compaction with a force of 600 kN (Figure 1b), followed by hot compaction with the same force (Figure 1c). Samples were heated to 420 ± 1 °C for 20 ± 0.1 min before hot compaction. Foaming took place at a temperature of 640 ± 1 °C for 15 ± 0.1 min, Figure 1d. The furnace used for heating was the Demiterm Easy 9 (Estherm d.o.o., Novaki, Croatia). The mould had a diameter of 4 ± 0.1 cm. A detailed explanation of the foaming process was described in a previous article [44]. Samples were cut in the dimensions: 2 × 2 × 2 ± 0.1 cm.

**Figure 2:**
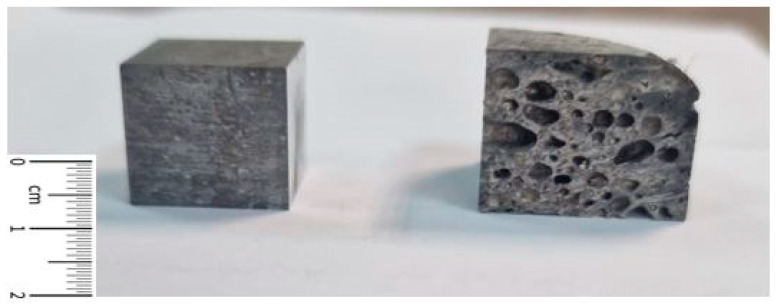
Bulk material and metal foam.

Design Expert 13 software from StatEase Inc. (Minneapolis, MN, USA) was used in this study to design an experimental plan and to statistically analyse the obtained results. The experimental design was based on a user-defined model. It was used to determine the effect of ageing temperature and time on the microhardness values of foams and bulk material (Figure 2). The input factors were determined by the user. Mathematical models and response surfaces were created based on the measured values.

The heat treatment was performed on all 20 samples. Before the first heating step, the samples of the base material were annealed to reduce the influence of the prior heat treatment.

The material was purchased in pre heated condition. The annealing was carried out at a temperature of 470 ± 1 °C and a duration of 3 ± 0.02 h, followed by cooling in the furnace. Foams did not go through this process as they melted during foaming, and the effects of prior heat treatment were thus removed. All samples were then subjected to a solution heat treatment at a temperature of 500 ± 1 °C. The duration of the solution heat treatment was 1.5 ± 0.02 h. The samples were then quenched in water. The next step was artificial ageing. Used temperatures were 150 ± 1, 175 ± 1, and 200 ± 1 °C. The ageing times were 2 ± 0.02, 9 ± 0.02, and 16 ± 0.02 h. The heat treatment parameters are listed in Table 2.

After heat treatment, the microhardness was measured. It was concluded from the previous studies that foaming temperature and density have no influence on the microhardness values [45]. Three microhardness measurements were performed on each sample using the Shimadzu HMV 2T tester (Shimazdu d.o.o., Zagreb, Croatia), with the value HV1 and the mean values were calculated. Prior to measurement, samples were sanded on 800 grit paper and polished. Microhardness values were measured 10 times on each sample, and the arithmetic value was given.

The next step was to analyse the microstructure through metallographic testing. After polishing, the surface was eroded to develop a microstructure. Etching agent was ordinarily used for microstructure development purposes of the aluminium alloys. It consisted of 0.5 ± 0.1 mL of 40% HF (Hydrofluoric Acid) dissolved in 100 ± 1 mL of water, at room temperature. Erosion time for each sample of foam and bulk material was 40 ± 1 s. The microscope used in the process was an OPTON Axioscope light microscope (EDC d.o.o., Zagreb, Croatia). This analysis was used to determine the difference between bulk material and foam with the various heat treatment parameters.

## 3. Results

The initial microhardness of the bulk material was 67 ± 2 HV1. The values of the foam fluctuated between 60 ± 2 and 90 ± 2 HV1. This could be due to the oxides on the surface. They are formed by the heat treatment during foaming and are mainly present in the surface layer where the microhardness was measured. They are also visible on the machining chips after the milling process. During compaction, the oxide layer breaks up and expands in all areas of the future foam. The oxides increase the microhardness values. It can therefore be concluded that foam can have higher microhardness values than the base material. It is also important to mention the foaming agent. While decomposing, its elements combine with the elements of the aluminium alloy and new chemical compounds are formed. This is particularly important when higher values of foaming agent are used. The oxides are useful because they act as stabilisers and promote pore roundness and homogeneous structure. Other samples that underwent heat treatment did not show variance in microhardness values on the surface. That is because with solution treatment, the structure became more homogeneous.

Table 3 shows the mean values of microhardness after heat treatment. It can be seen that there is a difference in the microhardness at different temperatures and the duration of the treatment.

The obtained results of the microhardness values are registered in Design Expert and then statistically analysed. Table 3 shows the values for bulk material. The used model was Quadratic, as suggested. The main parameters and their interconnection that were used are: A, which is a temperature of ageing, B as the time of ageing, AB and A^2^. The remaining parameters were excluded because the *p*-values were higher than 0.05. Values greater than 0.1 indicate that model terms are not significant. From the Table 4, it can be seen that the difference between Adjusted and Predicted R^2^ is less than 0.2, which is important proof that the model is adequate. Adeq Precision needs to be greater than 4, and is 50.2925 in this paper.

The model shows the influence of ageing temperature and time, and their interaction.

(1) $$\begin{array}{c} {Microhardness\;\left(bulk\;material\right)}^{-1.53}=0.005756-0.000061\times Ta-0.000107\times ta\\ +6.72378\times 10^{-7}\times Ta\times ta+1.73677\times 10^{-7}\times {Ta}^{2} \end{array}$$

Table 5 shows the parameters of the aluminium foam analyses. In this case, the model was Linear. The main parameters and their relationship that were used are: A as the ageing temperature, and B as the ageing time. The remaining parameters were excluded as in the previous model because the *p*-values were higher than 0.05. The F-value of the model, 8.08, means that the model is significant. The *p*-value shows that the probability of such a large F-value occurring due to noise is 1.52. The lack of fit is not significant, which is good because it is proven that the chosen model fits. The values of Predicted and Adjusted R^2^ differ by less than 0.2. The value of Adeq precision is greater than 4, which is desirable.

The equation which is given in this model can be written as:

(2) $$Microhardness\left(foam\right)=272.53667-0.800667\times Ta-2.15\times ta$$

## 4. Discussion

**Figure 3:**
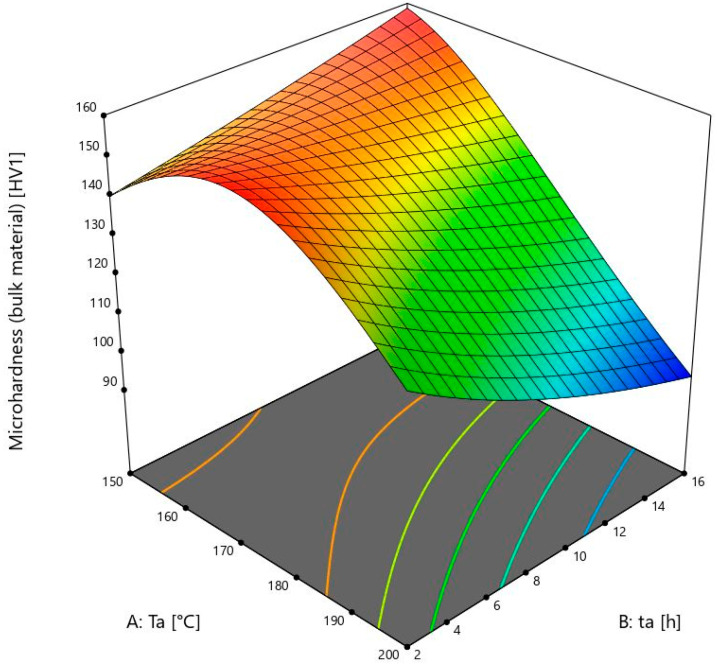
Microhardness model of the bulk material.

Figure 3 shows a 3D model of the microhardness of the bulk material.

The lowest ageing temperature in this study is 150 ± 1 °C. The samples of the bulk material show an increase in microhardness when the ageing time is increased from 2 ± 0.02 to 16 ± 0.02 h. The microhardness values change from 138 to 157 ± 2 HV1, which is much higher than the initial 67 ± 2 HV1. There is a lower increase of microhardness from 9 ± 0.02 to 16 ± 0.02 h than from 2 ± 0.02 to 9 ± 0.02 h. From this, it can be concluded that the values after 16 ± 0.02 h would soon start to decrease because of the visible reduction in microhardness growth within that time. This is also one of the maximum values after heat treatment. It is comparable to the value obtained at 175 ± 1 °C and the time of 2 ± 0.02 h, which was only 1 HV1 higher. It can be seen that the microhardness values of the bulk material in the model increase up to 175 ± 1 °C. After this temperature, the values are lower, or they start to fall in a shorter period of time.

At 175 ± 1 °C, the microhardness values of the bulk material decrease from sample 5, which is 158 ± 2 HV1, to sample 4 with a value of 129 ± 2 HV1. It is obvious that the microhardness values from 150 ± 1 °C to 175 ± 1 °C are still increasing, in contrast to the foam values. The microhardness at this temperature starts to decrease after heating for more than 2 ± 0.02 h. For further investigation, more times and temperatures should be chosen to obtain complete information, and the model would then be even more accurate. The ageing temperatures above 175 ± 1 °C are not useful because they would not give higher microhardness values, and the energy consumption would be higher, thus making the whole process more expensive.

The results obtained are consistent with the article [11], according to which the hardness of the solid material increases with time and then begins to decrease. After a certain time, the intermetallic compounds age and coagulate.

**Figure 4:**
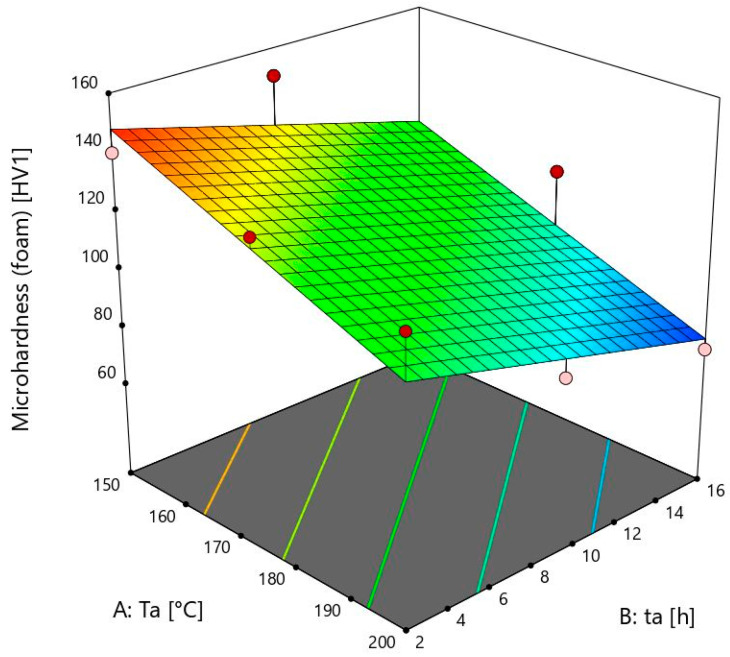
Microhardness model of the foam.

The model for the prediction of microhardness properties of aluminium foam is shown in Figure 4. The peak value at the ageing temperature of 150 ± 1 °C and ageing time of 9 ± 0.02 h is not visible because of the linear model.

Foam’s microhardness values are irreversible to the bulk material at 150 ± 1 °C. They decrease when the time reaches 16 ± 0.02 h, while the bulk material values increase. This is shown by sample 13, where the microhardness value is 114 ± 2 HV1. The rise of microhardness is visible from sample 12, which is subjected to ageing for 2 ± 0.02 h, with a microhardness of 140 ± 2 HV1, to sample 11 with a microhardness of 150 ± 2 HV1. These two values are similar to sample 13. For foams with a heat treatment temperature of 150 ± 1 °C, longer times such as 16 ± 0.02 h are not necessary. At this temperature, there is a need to heat for longer than two hours to obtain maximum microhardness. After 9 ± 0.02 h, precipitates that are desirable start to coagulate, and microhardness values start decreasing. In future work, more ageing times and temperatures will be used to obtain complete information on the maximum microhardness values at each ageing temperature. In contrast to the bulk material, the values of the foams at 150 ± 1 °C are much higher than the others. An exception is the time of 16 h, which can be exceeded after only 2 h at the temperatures of 175 ± 1 and 200 ± 1 °C. Temperatures under 150 ± 1 °C must therefore be taken into account when measuring foams’ microhardness. Unlike the bulk material, it can be seen that the values are decreasing with an increase in the ageing temperature. This is probably due to oxides and other compounds on the surface of the foams, which have a negative effect on the microhardness at higher ageing temperatures and times.

At a temperature of 175 ± 1 °C, the foam values decrease from 2 h to 16 h. With increasing duration from 9 ± 0.02 to 16 ± 0.02 h, the values decrease slightly, but with increasing duration from 2 ± 0.02 to 9 ± 0.02 h, the decrease of microhardness is much stronger. The maximum microhardness was obtained at 150 ± 1 °C, and unlike the bulk material, any higher temperature leads to a decrease in these values.

At 200 ± 1 °C, a deterioration in microhardness occurs. This is due to over-ageing. The microhardness of the bulk material decreases from sample 9 with 129 ± 2 HV1 to sample 10 with 93 ± 2 HV1 when ageing time increases from 2 ± 0.02 to 16 ± 0.02 h. The microhardness of foams decreases from 124 ± 2 HV1 to 74 ± 2 HV1. Values of foams and bulk material are similar, except at the time of 16 ± 0.02 h. It is proven that the foam’s microhardness is 20% lower than that of the bulk material at this time. This may be due to the presence of oxides, which, together with the coagulation of the precipitates, further deteriorate the microhardness over such a long period of time. The longest time for this temperature is 2 ± 0.02 h to improve results. In comparison, the microhardness of bulk material at 200 ± 1 °C and 16 ± 0.02 h is only slightly higher than the microhardness of the initial bulk sample. This is related to the article [35]. It shows how temperatures from 180 to 280 degrees lead to a decrease in hardness. It almost corresponds to the initial hardness before heat treatment, which is 65 ± 2 HV1.

As far as the foam is concerned, the last sample microhardness value is smaller than the initial foam microhardness. It can be concluded that there is no need for a high temperature and duration of ageing heat treatment. It is also associated with higher cost of energy and time, and there are no advantages regarding to increase in microhardness values.

### 4.1. Optimisation of the Parameters

Design Expert software was used to numerically optimise the microhardness of the foam and the bulk material. As the main objective, maximum microhardness of the foam was used with an importance of 5 out of 5. Microhardness of the bulk material was chosen with an importance of 3 out of 5. The ageing temperature and duration were chosen to be as low as possible in order to save energy and costs of the process. The temperature importance was 3, and the time was 4. Table 6 shows the optimum solutions for these parameters. It can be seen that the minimum ageing temperature chosen for this study gives the best results for the highest microhardness. The optimum times were also the lowest. The differences between the maximum value of the microhardness of the bulk material and the optimal solutions are below 15 HV1. Regarding foams, they differ from experimental less than 5 HV1. The deviation from the maximum value for both foam and bulk material is slightly minimal when the optimal time is lowest. This is important because the energy and time costs are the lowest. The foam has its maximum values at 9 ± 0.02 h and 150 ± 1 °C. That is much more time than 2 ± 0.02 h. In order to achieve only slightly higher microhardness, we would have to spend much more time.

**Figure 5:**
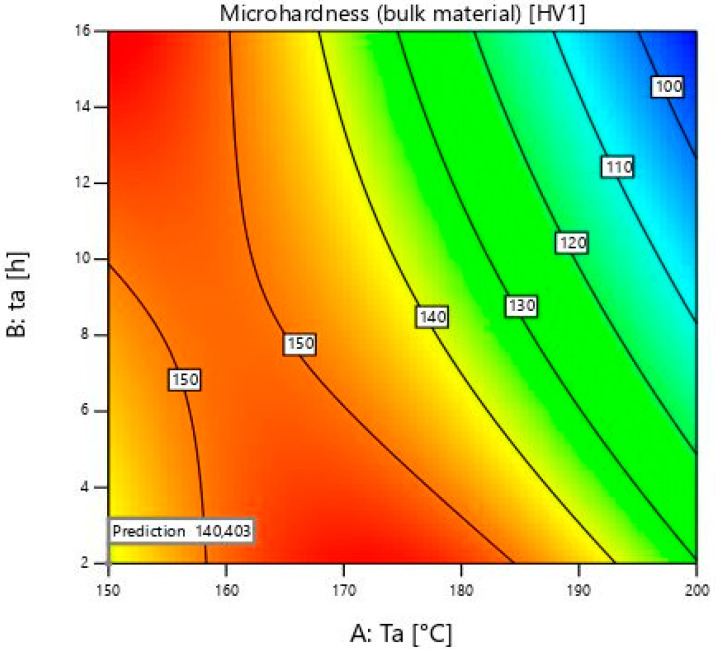
Diagram of optimal solutions for the bulk material.

Figure 5 shows a diagram with the optimal solutions for the specified numbers of importance. The red colour covers the highest microhardness values. It is visible from 160 to 180 ± 1 °C. It expands from 2 ± 0.02 to 16 ± 0.02 h. After 180 ± 1 °C, the colour of the diagram changes from yellow to blue. In that area, microhardness values decrease. The blue colour indicates the minimum values that are obtained at 200 ± 1 °C for 16 ± 0.02 h.

**Figure 6:**
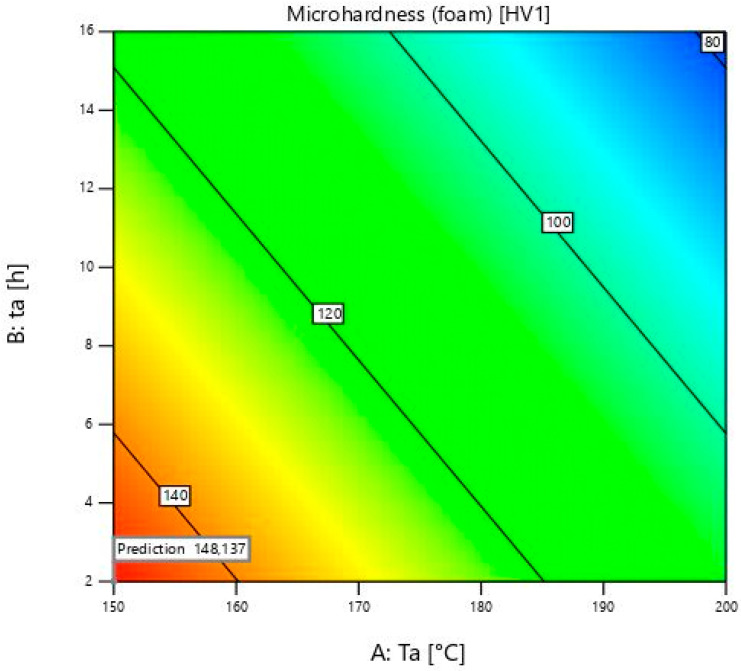
Diagram of optimal solutions for the foam.

Figure 6 shows the diagram of the optimum solutions for the aluminium foam. It differs significantly from the diagram for the bulk material. It can be seen that the red colour is only seen in a small area. It is located at the very bottom, at the beginning of the diagram. The maximum microhardness is reached at 150 ± 1 °C and a close time of 2 ± 0.02 h, which is also specified as the optimum value in Table 5. After that, microhardness values decrease and the colour changes from yellow to blue. The lowest values in the blue range are close to 200 ± 1 °C, and the maximum of heat treatment time, just as with the bulk material.

### 4.2. Microstructure of Samples

GP zones are small and round clusters with mostly zinc, magnesium atoms, and also fewer atoms of copper within the aluminium matrix. The presence of small precipitates affects the increase in hardness and strength after the heat treatment process. During heat treatment of the alloy EN AW 7075, a uniform microstructure with a fine aluminium matrix and dispersed secondary phases MgZn_2_ is formed. These metastable phases form primarily at lower temperatures through the connection of small GP zones. This leads to a significant increase in microhardness. This was also noted in the article [46]. At lower temperatures, such as 105 degrees, a longer ageing period is required to achieve the same results as at a temperature of 120 degrees.

As mentioned in the article [12], the values of microhardness are related to the intermetallic compounds MgZn_2_ that are formed during heat treatment. If the microstructure is cooled slowly after annealing, a coarse grain is formed, which reduces the hardness. The same applies to a long holding time at the ageing temperature. The even distribution of these compounds also contributes to better material stability and higher hardness.

Larger visible particles with uneven shape are oxides and intermetallic inclusions that have coagulated in relation to fine precipitates can also be seen with a larger duration. Dislocations, microcracks and excretion of intermetallic compounds along the grain boundaries may be present in the structure of the bulk material, but less than in the foam structure. With a longer duration of heat treatment, larger, stable particles can be seen in the material. They decrease microhardness after heat treatment.

**Figure 7:**
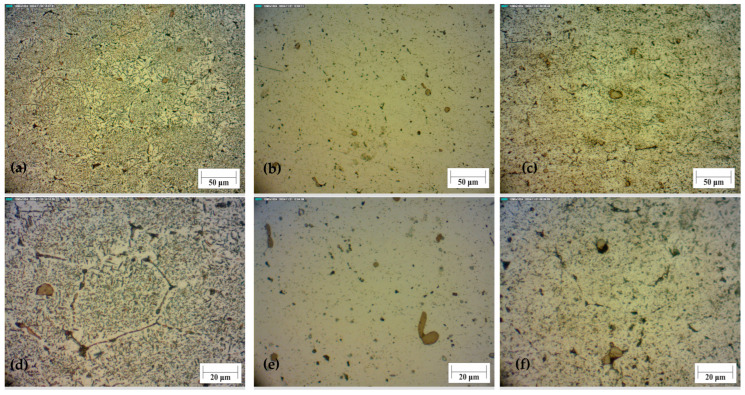
Microstructure of the bulk alloy under a magnification of 200× (**a**–**c**) and 500× (**d**–**f**). (**a**,**d**) non-treated samples; (**b**,**e**) sample 5; (**c**,**f**) sample 8.

In Figure 7, the microstructure of the bulk material before and after heat treatment can be seen. Figure 7a shows the bulk material without heat treatment. Many intermetallic compounds can be seen. In comparison, sample 5 has the highest microhardness values. The microstructure of this sample is with a more visible aluminium matrix with a smaller number of precipitates. They are finely dispersed in the matrix, with only a few larger agglomerates, Figure 7b,e. That homogeneous structure enabled a rise in microhardness. One of the samples with the lowest microhardness values is sample 8 with ageing at 200 ± 1 °C and a period of 9 ± 0.02 h. The structure of that sample is similar to the untreated sample. There is a large number of precipitates, and some of them have coagulated. With additional time, this structure would be the same as the untreated. The light area, which represents α aluminium matrix, would be less seen. Because of the high temperature and longer time, there are many more precipitates than in sample 5, and they are not evenly distributed.

Compared to the metallographic images from the article [11], which show the structural change at 190 degrees, it is obvious that there is more precipitation at the same ageing time compared to Figure 7e. The reason for this is that the process is faster at higher temperatures, which is why the maximum is at a lower time.

**Figure 8:**
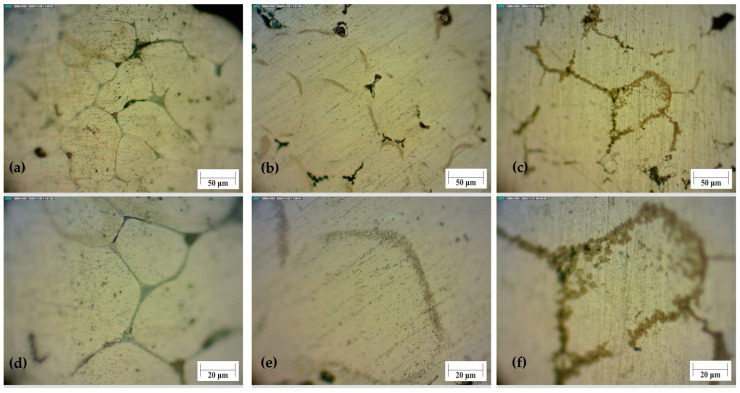
Microstructure of the foam under a magnification of 200× (**a**–**c**) and 500× (**d**–**f**). (**a**,**d**) non-treated samples; (**b**,**e**) sample 11; (**c**,**f**) sample 18.

The foam sample without any treatment has visible cracks at the boundaries of the grain. It also has a coarse structure in comparison to the bulk material. This is due to the production of foams from machining chips. Some parts of the material did not melt during foaming, because of the oxides that rise to this temperature. If the foaming agent decomposes in the solid material, this material will have cracks. That crack can be seen at the untreated sample’s surface as the black area, Figure 8a. Heat treating the foam at a temperature of 150 ± 1 °C, intermetallic phases break into fine precipitates, and microhardness increases above 50%. After ageing treatment at 150 ± 1 °C and 9 ± 0.02 h, intermetallic compounds start to nucleate and coarsen. The difference between the foam structure and the structure of the bulk material is the position of the precipitates. The microstructure of the base material, as seen in optical microscopy, is comparable to that of the article [33]. The compounds occur within the grain boundaries and are more evenly distributed. It was observed that an increase in cooling rate leads to a decrease in grain size and an increase in properties, while in this article, the dependence of properties on time is observed.

Before and after the heat treatment of aluminium foam, intermetallic compounds in the structure of foams are visible at the grain boundaries, Figure 8. Some pores can also be seen as dark areas. The difference between samples 11 and 18 is in the size of precipitates, Figure 8e,f. The microhardness of sample 11 was the highest. That is proven with small precipitates at grain boundaries. Sample 18 has larger precipitates, due to the higher temperature of the heat treatment. These precipitates started to coagulate, and the microhardness values decreased.

This structure is similar to that shown by optical microscopy in the article [41]. Intermetallic compounds occur at the grain boundaries and grow with increasing temperature and ageing time. The hardness values at 180 degrees start to decrease faster and reach the initial values, as in this case, at 200 degrees. At lower temperatures than 150 degrees, a maximum is reached for both items.

### 4.3. SEM/EDS Analysis

**Figure 9:**
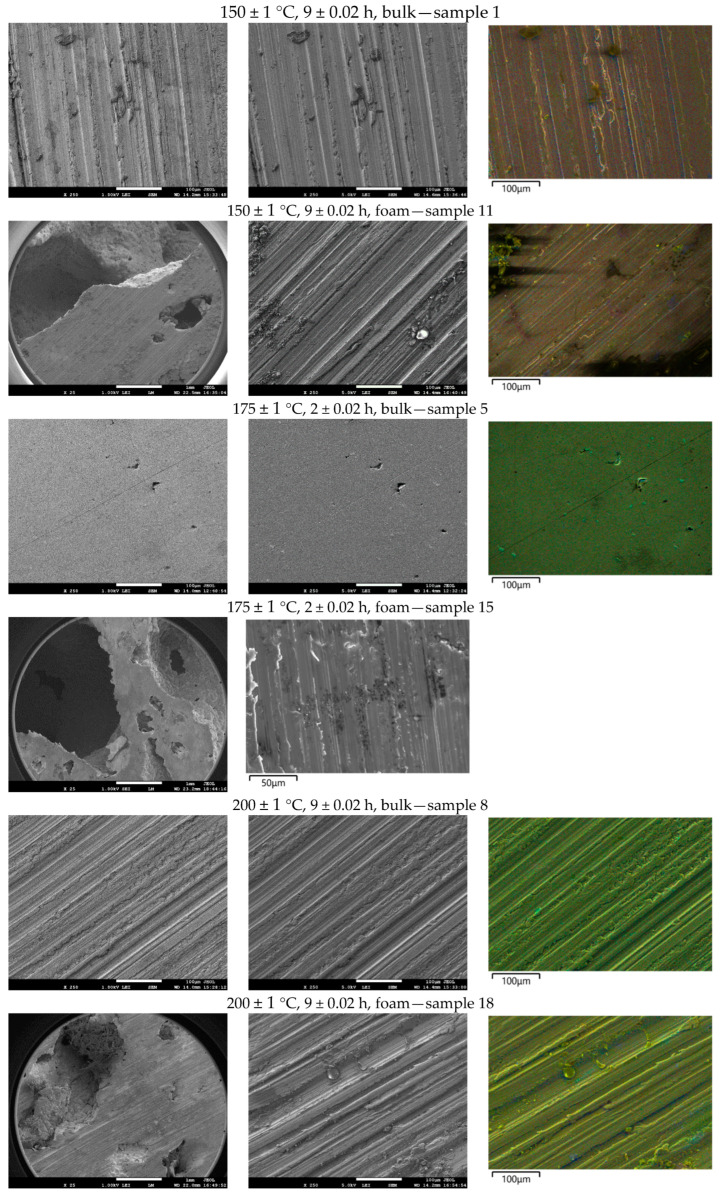
SEM images of selected samples.

The morphology and elemental analysis of the selected samples (1, 11, 5, 15, 8 and 18) were characterised using a scanning electron microscope (SEM) model JEOL JSM-7610FPlus (JEOL, Tokyo, Japan). The SEM used was equipped with an Oxford Instruments AZtec Live ADVanced UltimMax 65 EDS detector (Oxford Instruments, Abingdon, UK) to perform the elemental composition analysis (EDS). The EDS spectra obtained were analysed using Oxford Instruments AZtec software platform ver. 6.0 (Abingdon, UK). The EDS analysis was performed on the surface of three solid samples and three foam samples. An EDS map with a resolution of 4096 × 2816 pixels was created (each pixel is a separate EDS spectrum). Samples of foam and solid material were selected for which the highest and lowest hardness was measured. Figure 9 shows SEM images of the selected samples.

Table 7 shows the weight percentage of the most important elements in the selected samples. From the attached results, it can be seen that the content of Mg and Zn does not change significantly in the observed samples. However, a significant difference can be seen in the O content, particularly in the foam samples. This is to be expected, as oxidation of aluminium takes place during foaming. In addition, foams have a larger surface area than solid material, which allows the formation of more aluminium oxide compared to solid samples. Therefore, the proportion of oxygen in the chemical composition is greater.

The chemical composition of the foams also shows the presence of Ti, which is to be expected as TiH_2_ was used as a foaming agent. It is not present in the solid samples.

**Figure 10:**
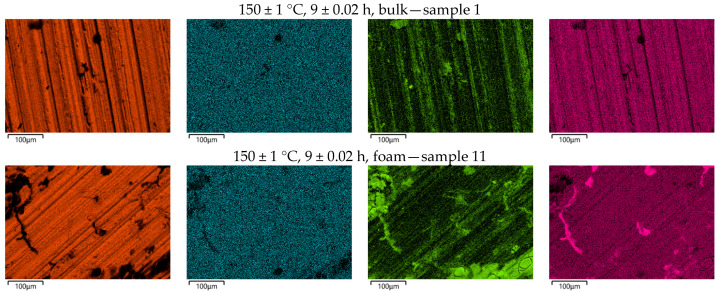

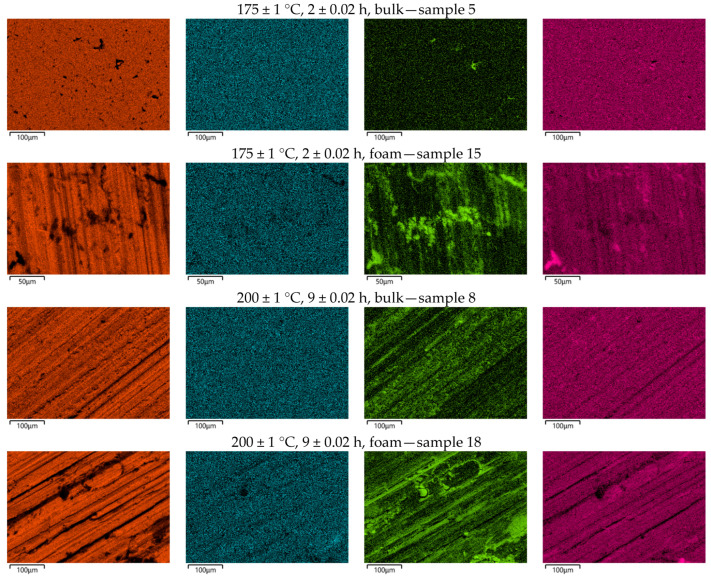
Element distribution on the surface of the selected samples.

Figure 10 shows the distribution of the elements on the tested surfaces. Comparing the EDS images of the foams and the solid samples, it is clear that the distribution of Mg and Zn on the surface of the samples is more or less uniform. However, it should be emphasised that there are certain differences. The best distribution can be seen in sample 5, which also has the highest hardness. This is a consequence of the near-optimal parameters of the subsequent heat treatment, which led to the formation of small and finely distributed precipitates.

The lowest hardness was measured in sample 18, which also had the highest oxygen content. Figure 10 shows a significant accumulation of oxides on the surface of sample 18, which is a direct consequence not only of the foaming but also of the long-term subsequent heat treatment of the maturation. This actually resulted in excessive ripening, which led to a reduction in hardness.

## 5. Conclusions

The main aim of this work was to determine the difference in microhardness values between heat-treated bulk material and foam with untreated samples. Modelling and optimisations were performed in Design Expert. Few conclusions were made:

- Aluminium foam has a higher microhardness than the base material before heat treatment. This is due to the oxides on the surface, which are created as a product of foaming at higher temperatures and using machining chips that have oxides on the surface.
- The highest microhardness value for bulk material is reached at 175 ± 1 °C and a duration of 2 ± 0.02 h, as well as at 150 ± 1 °C and a duration of 16 ± 0.02 h. Values were 158 ± 3 and 157 ± 2 HV1. The microhardness increases up to 175 ± 1 °C at 9 ± 0.02 h. The maximum value could be between 2 ± 0.02 and 9 ± 0.02 h.
- Foam maximum microhardness was reached at the lowest temperature in this study, at 150 ± 1 °C and the time of 9 ± 0.02 h. The maximum value was 150 ± 2 HV1. From the results, it is suggested that the maximum microhardness value for this EN AW 7075 alloy foam can be achieved at lower temperatures. This is due to the fact that the values of microhardness decrease as the temperature increases. Further investigation should be carried out to confirm this statement.
- The model of bulk material is Quadratic because the microhardness values increase and decrease at higher ageing temperatures, while the foam model is Linear because the values only decrease with increasing ageing temperature and time.
- An optimisation was carried out in Design Expert. Optimal values of microhardness were at the lowest ageing time of two hours and the lowest ageing temperature of 150 ± 1 °C. This is because a lower duration is considered more important, and also the maximum microhardness of the foam value. It is only a small difference between the highest foam value and the optimal value, but the duration is much lower. In that way, there are higher energy and cost savings while heat treating an aluminium foam.
- Metallographic figures showed the difference between the foam and the bulk material. The precipitates in the bulk material were dispersed in the matrix, and some of them were on the grain boundaries. The foam had precipitates mostly at the boundaries of grains. The samples with heat treatment at 200 ± 1 °C showed a larger number of precipitates with larger size in the bulk material, and growth of precipitates in the foam. That is the reason for the microhardness decrease.
- The SEM/EDS analysis confirmed the previous assumptions as to why the hardness values presented here were achieved. The significant presence of oxygen in the foam samples was detected, and the EDS analysis showed its distribution.

This research leads to an improvement in the foam’s microhardness. In further research, the strength and energy absorption values will be included with more heat treatment temperatures and times. It is important to decrease the temperature to see if the foam’s maximum values will be achieved.

## Figures and Tables

**Table 1:** Chemical composition of the alloy EN AW 7075 [43].

Zn	Mg	Cu	Fe	Si	Mn	Cr	Ti
5.1–6.1	2.1–2.9	1.2–2	max 0.5	max 0.4	max 0.3	0.18–0.28	max 0.2

**Table 2:** Heat treatment parameters.

Sample Number	Aging Temperature (°C)	Aging Time (h)
1 (bulk material)	150 ± 1	9 ± 0.02
2 (bulk material)	150 ± 1	2 ± 0.02
3 (bulk material)	150 ± 1	16 ± 0.02
4 (bulk material)	175 ± 1	16 ± 0.02
5 (bulk material)	175 ± 1	2 ± 0.02
6 (bulk material)	175 ± 1	9 ± 0.02
7 (bulk material)	175 ± 1	9 ± 0.02
8 (bulk material)	200 ± 1	9 ± 0.02
9 (bulk material)	200 ± 1	2 ± 0.02
10 (bulk material)	200 ± 1	16 ± 0.02
11 (foam)	150 ± 1	9 ± 0.02
12 (foam)	150 ± 1	2 ± 0.02
13 (foam)	150 ± 1	16 ± 0.02
14 (foam)	175 ± 1	16 ± 0.02
15 (foam)	175 ± 1	2 ± 0.02
16 (foam)	175 ± 1	9 ± 0.02
17 (foam)	175 ± 1	9 ± 0.02
18 (foam)	200 ± 1	9 ± 0.02
19 (foam)	200 ± 1	2 ± 0.02
20 (foam)	200 ± 1	16 ± 0.02

**Table 3:** Microhardness values.

Sample Number	Microhardness (HV1)
1 (bulk material)	153 ± 2
2 (bulk material)	138 ± 2
3 (bulk material)	157 ± 2
4 (bulk material)	129 ± 2
5 (bulk material)	158 ± 2
6 (bulk material)	139 ± 2
7 (bulk material)	142 ± 2
8 (bulk material)	110 ± 2
9 (bulk material)	129 ± 2
10 (bulk material)	93 ± 2
11 (foam)	150 ± 2
12 (foam)	140 ± 2
13 (foam)	114 ± 2
14 (foam)	101 ± 2
15 (foam)	132 ± 2
16 (foam)	107 ± 2
17 (foam)	107 ± 2
18 (foam)	87 ± 2
19 (foam)	124 ± 2
20 (foam)	74 ± 2

**Table 4:** Analyses of variance of bulk material.

	F-Value		*p*-Value
Model	276.98		<0.0001
A-T_a_	591.58		<0.0001
B-t_a_	143.83		<0.0001
AB	246.58		<0.0001
A^2^	125.91		<0.0001
Lack of Fit	1.73		0.5101
Std. Dev.		0.0000	
Mean		0.0006	
C.V. (%)		2.59	
R^2^		0.9955	
Adjusted R^2^		0.9919	
Predicted R^2^		0.9722	
Adeq Precision		50.2925	
PRESS		6.949 × 10^−9^	
−2 Log Likelihood		−200.72	
BIC		−189.21	
AICc		−175.72	

**Table 5:** Analyses of variance of foam.

	F-Value		*p*-Value
Model	8.08		0.0152
A-Ta	10.32		0.0148
B-ta	5.83		0.0464
Lack of Fit	44.2		0.1146
Std. Dev.		15.26	
Mean		113.07	
C.V. (%)		13.5	
R^2^		0.6977	
Adjusted R^2^		0.6113	
Predicted R^2^		0.4261	
Adeq Precision		8.3896	
PRESS		3095.5	
−2 Log Likelihood		79.32	
BIC		86.23	
AICc		89.32	

**Table 6:** Optimal heat treatment parameters.

Number of Optimization Solution	Aging Temperature [°C]	Aging Time [h]	Microhardness (Bulk Material) [HV1]	Microhardness (Foam) [HV1]
1	150	2	140	148
2	150	2.3	141	148
3	152	2	143	146
4	155	2	146	145

**Table 7:** wt.% of most important elements in selected samples and associated colours.

Element	Al [wt.%]	Mg [wt.%]	O [wt.%]	Ti [wt.%]	Zn [wt.%]
EDS color					
Sample 1	84.8 ± 0.1	2.7 ± 0.1	3.3 ± 0.1	0.0 ± 0.1	5.8 ± 0.1
Sample 11	79.1 ± 0.1	2.3 ± 0.1	8.8 ± 0.1	0.3 ± 0.1	5.7 ± 0.1
Sample 5	86.9 ± 0.1	2.5 ± 0.1	0.6 ± 0.1	0.0 ± 0.1	6.0 ± 0.1
Sample 15	81.3 ± 0.1	2.6 ± 0.1	7.7 ± 0.1	0.4 ± 0.1	5.7 ± 0.1
Sample 8	83.9 ± 0.1	2.5 ± 0.1	4.1 ± 0.1	0.0 ± 0.1	5.7 ± 0.1
Sample 18	76.4 ± 0.1	2.5 ± 0.1	11.4 ± 0.1	0.5 ± 0.1	5.5 ± 0.1

## Data Availability

The original contributions presented in the study are included in the article, further inquiries can be directed to the corresponding author.
